# How to debrief teamwork interactions: using circular questions to explore and change team interaction patterns

**DOI:** 10.1186/s41077-016-0029-7

**Published:** 2016-11-15

**Authors:** Michaela Kolbe, Adrian Marty, Julia Seelandt, Bastian Grande

**Affiliations:** 1 University Hospital Zurich, Simulation Center, Rämistrasse 100, 8091 Zurich, Switzerland; 2 ETH, Zurich, Switzerland; 3 University Hospital Zurich, Institute for Anaesthesiology, Rämistrasse 100, 8091 Zurich, Switzerland; 4 University Hospital Zurich, Quality Management and Patient Safety, Rämistrasse 100, 8091 Zurich, Switzerland

**Keywords:** Debriefing, Teamwork, Circular question, System, Team interaction pattern

## Abstract

We submit that interaction patterns within healthcare teams should be more comprehensively explored during debriefings in simulation-based training because of their importance for clinical performance. We describe how *circular questions* can be used for that purpose. Circular questions are based on social constructivism. They include a variety of systemic interviewing methods. The goals of circular questions are to explore the mutual dependency of team members’ behavior and recurrent behavior patterns, to generate information, to foster perspective taking, to “fluidize” problems, and to put actions into relational contexts. We describe the nature of circular questions, the benefits they offer, and ways of applying them during debriefings.

## Introduction

In the OR, do you feel that your colleagues share their points of view more frequently before or after the attending enters the OR?

Who can typically speak up the most to the attending when she seems stuck in unsuccessfully performing an unexpected difficult intubation?

These questions differ from the questions that we usually ask during debriefings, and they certainly differ from questions we use in our daily conversations. They are called *circular questions* [1–3]. Originally developed in systemic family therapy, they are useful for exploring and changing learners’ potentially gridlocked explanations of team interactions during debriefings [4–6].

In this manuscript, we describe the nature of circular questions, the benefits they offer, and ways of applying them during debriefings. Debriefings—the instructor-guided conversations among learners aiming to reflect on the relationships among events, actions, thought and feeling processes, and performance outcomes of the simulation—play a crucial role during simulation-based training [7–10]. Notably, we do not consider circular questions a new debriefing method on its own; instead, we regard them as an extension to any debriefing method aiming at making debriefings even more effective by helping learners gain new perspectives and understand teamwork patterns.

## Debriefing team interactions

Teamwork and its components (e.g., leadership, communication) are typical content of learning objectives in simulation-based training [4, 5, 11–20]. This is important because effective teamwork has been linked to patient safety [21, 22]. Some teamwork components are attitudinal such as team orientation (i.e., preference for working with others) while others are behavioral and process-like such as leadership and mutual performance monitoring [23]. Simulation-based training mostly addresses the latter. Team process is defined as “members’ interdependent acts that convert inputs to outcomes through cognitive, verbal, and behavioral activities directed towards organizing taskwork to achieve collective goals” (p. 357) [24]. From our view, this team process, the interdependency of team members’ actions, and their importance for clinical performance are not yet fully explored during debriefings.

First, discussions during debriefings tend to be focused on team members’ *individual* behaviors and frames about their *individual* actions [25]. This focus is important for uncovering the reasoning behind individual actions [9, 26]. Yet, it is potentially not sufficient for surfacing team dynamics because it does not yet allow for uncovering mutual dependency of behavior or self-reinforcing behavior patterns. Exploring these patterns would be beneficial: recent research has shown that it is particularly the pattern of behaviors among team members that discriminate higher from lower performing teams [27–32]. For example, during inductions of general anesthesia, higher-performing teams showed more talking aloud patterns than average-performing teams. [27] Also, teams engage in solution-oriented as well as complaining-oriented sequential patterns which are associated with positive and negative team mood, respectively [33, 34]. So far, these team interaction patterns have rarely been explicitly uncovered, explored, or altered in debriefings, resulting in missed opportunities for new information, perspectives, and change.

Second, by focusing on individual thought and feeling processes rather than on team interactions, instructors might overestimate the individual’s capacity and underestimate the influence of the context on the individual. Thus, we make, as Ross [35] described, the *fundamental attribution error*: we overestimate the individual learner’s disposition and personality and underestimate their environment and situational dynamics. With respect to teamwork, this means that instructors might overestimate the *linear causality* of teamwork behaviors and underestimate the *circular causality* of behaviors, that is the mutual dependency of behaviors; a person’s behavior at one time is considered both effect and cause of the interactional partner’s behavior [36]. For example, while the assumption that *the more internally willing individual team members were to speak up, the more effort they would put into actually speaking up* would be based on linearity; the assumption that *if team member A speaks up and team member B reacts with verbal appreciation, team member A might feel encouraged to speak up in future teamwork* would be based on circularity. So far, circularity is rarely systematically explored during debriefings, missing an essential element of teamwork.

Third, using linear rather than circular assumptions, we tend to underestimate the meaning and messages of feelings, thoughts, and actions within the team as *system*. For example, by asking

Why didn’t you speak up? – (Learner: “I don’t know.”)

not much is yet explored about the potential function of *not* speaking up for the system such as preserving hierarchy within the team, maintaining responsibilities or respecting sub-team territory [3, 18]. The resulting lack of new, different information tends to have a conservative effect on the learner, which may implicitly validate preexisting beliefs (e.g., that speaking up is too risky) [2]. As a further result, instructors may stop exploring an issue in more detail because they feel that they have understood “enough”. This phenomenon has been called the confirmation bias, that is, seeking or interpreting information in a way that confirms existing beliefs, expectations, and hypotheses [37]. Instructors are then at risk to develop solutions that do not fit the (not yet comprehensively discovered) problem [38].

In sum, we submit that circular interactional patterns within healthcare teams and their importance for clinical performance should be explored more comprehensively during debriefings. We propose that by applying systemic thinking and asking circular questions, instructors can actively use debriefings to walk the talk of teamwork.

## The nature of circular questions

Circular questions are based on social constructivism and on circular assumptions about an issue [2, 39]. They were developed by the Milan Associates, a group of family therapists that fundamentally advanced the field of systemic family therapy [1]. They described circular questions as an interviewing tool to explore a relationship between two people as it is seen by a third person by—in a somewhat complex way—inviting the third person to describe the relationship of two others in their presence. For example, a daughter is asked to describe how she sees the relationship between her sister and her mother or how everybody in the family reacts to a reported problematic behavior [1]. Meanwhile, the term circular questions subsumes a variety of systemic oriented interviewing tools with the goal of exploring recurrent patterns and processes, generating information, fostering perspective taking, “fluidizing” problems, and putting actions into relational contexts [3, 40–42]. More specifically, by using circular questions interactions are explored with respect to differences in *behavior* rather than personality traits, *ranking and classification*, change in the relationship *before and after an event*, and differences in respect to *hypothetical conditions*.

**Fig. 1:**
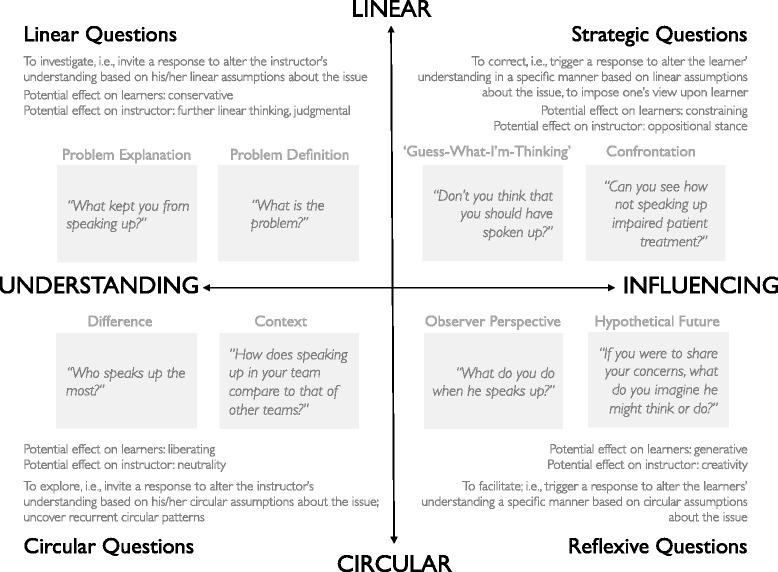
Four major types of questions based on the dimensions *assumptions* (linear vs. circular) and *intent* (understanding vs. influencing) based on Tomm [2, 43, 53] and others [9, 52, 54, 55] and respective objectives and examples for debriefings

To provide an overview, classify, and illustrate different types of questions, we used Tomm’s [2] framework and adapted it to healthcare simulation debriefings (Fig. 1). Tomm used two dimensions—assumptions and intent—to distinguish four types of therapeutic interview questions: linear, circular, strategic, and reflexive (Fig. 1) [2]. The *intent* dimension refers to intended locus of change, that is, whether to orient oneself or change the others [2]. The *assumptions* dimension refers to whether the instructor considers predominantly linear or circular assumptions about an issue [2]. According to Tomm, the intent of linear and circular questions is to orient oneself whereas the intent of strategic and reflexive questions is to change others [2]. Linear and strategic questions are based on linear assumptions whereas circular and reflexive questions are based on circular assumptions [2, 43]. Notably, most literature summarizes reflexive questions under *circular questions* and we follow that tradition [1–3, 40, 44–47]. Based on Tomm’s framework, in Fig. 1, we illustrate the use of these four types of questions in healthcare simulation debriefings. As these four types of questions differ in their intent and assumptions, they differ in their potential effect on both learner and instructor as well [2]. For example, whereas strategic questions may have a constraining effect on the learner and trigger the instructor to move into an oppositional stance to the learner, circular/reflexive questions may have a generative effect on the learner and help the instructor to be creative (Fig. 1) [2].

## Applying circular questions during debriefings

Notably, we do not consider circular questions a stand-alone debriefing method. Recent literature has highlighted the benefits of applying blended debriefing approaches [5, 6, 25, 48]. For example, the *Debriefing with Good Judgment Approach* can be used to identify frames that drive actions [9]. Circular questions can complement this approach by highlighting the diversity of assumptions within a team, exploring team dynamics, and by helping the team to develop systemic solutions. That is, we recommend adding circular hypothesizing and asking circular questions to the instructor’s debriefing toolbox and to embed them in a respectful and engaging context. Further considerations and requirements for incorporating circular questions are described in Table 1.

**Table 1:** Considerations and requirements for using circular questions during debriefings

Considerations and requirement	Details
Psychologically safe learning environment	Rudolph and colleagues have suggested a number of actions the instructor can take at the pre-briefing to establish a respectful and psychologically safe learning environment: for example clarifying mutual expectations, establishing a “fiction contract,” orienting to logistic details, and explicitly declaring and enacting a commitment to respecting learners and concern for their psychological safety [49].
Holding the learner in high regard	The “basic assumption,” as noted by Rudolph and colleagues, is an explicit statement to hold the learner in high regard: considering every participating learner intelligent, capable, doing their best, and wanting to improve [8].
Systemic assumptions about teamwork	Instructors benefit from (1) formulating hypotheses about team interaction patterns *(hypothesizing)*, (2) investigating these hypotheses based on reactions of the team to information about aspects such as meaning, difference, change, etc. *(circularity)*, and (3) triggering feedback and inquiring opinions rather than allying with specific team members *(multipartiality)* [1, 41].
Previewing	As circular questions can be unfamiliar to the instructors and learners, previewing them to explicitly orientate the learners to this method may enhance understanding and transparency. For example, “I’d like to understand you more and would like to ask you an unfamiliar type of question: …” [50, 56].
Balancing advocacy and inquiry	If circular questions are used excessively, the instructor becomes impalpable to the learners and they might get frustrated from lack of direction and disengage from the debriefing [52]. Learners will not only need to perceive the instructor as someone trustworthy but also as someone who is willing to share his or her thinking, point of view, and expertise [2, 9, 50].

### How to use circular questions in debriefings

In order to use circular questions during debriefings, the instructor needs to adopt a *systemic mindset*. One essential part of that mindset is based on what Rudolph and colleagues have also described in their *Debriefing with Good Judgment Approach* as holding the learner in high regard and combining honesty with curiosity [9, 49, 50], reflecting the systemic premises of respectfulness and curiosity [40]. Another part of the instructor’s systemic mindset is viewing teamwork as circular process, that is, considering team members’ behavior as mutually dependent, reflecting the systemic premise of circularity [1, 3, 47]. A third part of the systemic mindset is viewing teamwork perceptions as socially constructed, that is, taking into account that each team member has developed his/her own “truth” about teamwork, reflecting the systemic premise of social constructivism [39, 40]. As an example, consider the following simulation scenario:

A trauma patient arrived in the emergency department. While handing the patient off, there seems to be confusion, many voices are heard, each team member seems to be engaged in action. There is no structured information exchanged or verbal planning. The paramedic repeats himself frequently, getting louder each time.

Approaching this episode systemically, the instructor might consider the paramedic as competent and experienced in performing patient handovers (respect), be curious about how team members mutually influenced each other and the paramedic’s behavior (e.g., in response to what behaviors by all team members did the paramedic start repeating himself; circularity) and the differences and similarities in each team members’ perception of the handover (social constructivism).

Circular questions differ in their objectives: understanding the learners (i.e., explore existing frames with difference- and context-oriented questions) vs. explicitly helping learners to change (i.e., identify new frames with observer-perspective and hypothetical-future questions; Fig. 1). More detailed examples of the circular questions outlined in Fig. 1 are provided in Table 2.

**Table 2:** Examples of circular questions [1–3, 40, 42, 43, 45, 53, 54] adapted to debriefings

Question type^a^	Difference				Context	Observer perspective				Hypothetical future			
Objective	Understanding									Influencing			
Purpose	Explore how behavior varies according to contexts	“Fluidize” personality characteristics	Explore causal attributions and diverse views	Explore sequences	Explore meaning of behavior within a context	Become a better observer of oneself	Encourage “other” awareness	Explore interpersonal perception	Explore interpersonal interaction	Highlight potential consequences	Explore catastrophic expectations	Explore future action	Explore dilemmas
Examples	“When do your colleagues speak up most? … What is different in these situations?”	“What does she do when she does not seem to be interested in your opinion? … How do you explain that?”	“If he insists on doing the checklist, do you imagine he does this as a matter of principle or because he is convinced of its use in this situation?”	“In the OR, is there more speaking up before or after the attending joins the team?”	“How do you explain that she was shouting multiple instructions at the same time?”	“When you responded the way you did, how did you feel about your reaction?” “If an intern had observed your interaction, what do think he might have learned from you?”	“What do you imagine he experiences when he gets into a situation like that?”	“What does he think that you think is going on when he starts yelling?”	To A: “What do you do when she starts doing the checklist without everybody being present? … And when you do this, what does she do?” To B: “What do you do when he makes that comment? … And when you do this, what does he do?”	“If you continued not to talk about it, what do you expect would happen to the team?”	“What are you worried might happen if you said that you have never placed a central line before?”	“If she were saying ‘OK, I’ll take the lead’ when she is joining a resuscitation, what do you imagine the other team members would do?”	“If she were joining this critical situation as attending—do you imagine her first goal is to get an overview or distribute tasks among team members?”

^a^There is overlap among the types of question

### When to use circular questions in debriefings

From our experience, circular questions which facilitate understanding are more useful at the beginning of a discussion or when examining a specific debriefing topic. That is, they help surface the variety and diversity of frames, which are the invisible mental models that drive people’s actions [9, 26, 50], and behavior patterns. They may also foster perspective-taking. An example for a *context-oriented question* (Table 2) could be:

Hubert, when you hear Michael say that he has had these concerns during the scenario but did not voice them to Daniel, how do you explain this?

Circular questions which may facilitate change are more useful during later periods when working on solutions, for example, using an *observer-perspective question* (Table 2). An example could be:

Hubert, you were able to observe the interaction between Michael and Daniel. I’m curious about your perspective, what do you think Michael might have needed from Daniel to speak up in that situation?

Furthermore, there a number of healthcare simulation debriefing situations in which circular questions may be particularly useful. We explain them in the following paragraphs.

#### When the instructor feels s/he would like to learn something new and explore a topic/frame deeper

The concept of double-loop learning suggests that discovering and potentially changing frames leads to deep learning [9, 26, 50]. Exploring learners’ frames such as “I think that there are people who just don’t have a sense of responsibility” by asking *difference-oriented questions* (Table 2) might help reveal more facets of this frame. Examples of two questions which could expand thinking or trigger reflection:

From your perspective, what does someone do who has a sense of responsibility?

and following up with

[to more team members] What else [does someone do who has a sense of responsibility]?

and

From your point of view, are these actions something that can be shared by all team members or should they remain with the team leader?

#### When the instructor feels that s/he is taking sides

Instructors may sometimes feel that they are taking sides with a particular team member or professional subgroup within the team (e.g., doctors or nurses). Asking a *context-oriented question* (Table 2) to multiple team members might help the instructor to be less biased and show more understanding [46], for example:

If there was a good reason for not voicing your suggestions in this department, what would that reason be?

#### When the instructor feels that s/he is moving into an oppositional stance from the learners

Instructors may sometimes sense that they are moving into an oppositional stance from that of the learners. That is, instructors may feel the need to convince the learner to agree with him/her. This usually goes along with asking strategic questions (Fig. 1) such as “Don’t you think you should have spoken up?” [2, 8, 50]. Switching gears by asking question based on circular assumptions might offer more generative responses, for example, the *context-oriented question* above *and difference-oriented questions* (Table 2) such as

What was different in instances in which you did speak up?

#### When the instructor wants to highlight different points of view among team members

Especially *difference-oriented questions* (Table 2) can help surface the diversity of views and help develop team mental models, that is, a shared and accurate understanding of taskwork and teamwork [51]. Examples could be:

In the OR, do you feel that your colleagues speak up more before or after the attending comes in?

Who else do you think might think this way?

or

Who do you think might be more skeptical?

#### When the instructor wants to explore circularity among team members

Team dynamics and interaction patterns play a crucial role in teamwork [27–32]. *Observer-perspective questions* (Table 2) can help identify mutual dependency of behavior. Examples could be:

What do you do when she starts doing the checklist without everybody being present? […]

And when you do this, what does she do?”

*Context-oriented questions* (Table 2) can help explore how teamwork varies according to contextual conditions, for example:

How do you explain that he sometimes shouts multiple instructions at the same time in a trauma case?

## Discussion

In this manuscript, we have introduced circular questions as a way of debriefing team interactions. We have proposed that the interdependency of team members’ actions, and their importance for clinical performance should be more comprehensively explored during debriefings because (1) recent research has shown that rather than individual actions of single team members, it is the interaction pattern among team members that discriminate higher- from lower-performing teams [27–32], (2) by focusing on individual thought and feeling processes rather than on team interactions, instructors might overestimate the *linear causality* of teamwork behaviors and underestimate the *circular causality* of behaviors, and (3) by asking linear questions, instructors may tend to underestimate the meaning and messages of feelings, thoughts, and actions within a system, missing new information and team phenomena.

We have described the nature of circular questions and how they allow for exploring team behavior patterns, generating new information, and fostering perspective taking and observation skills in the debriefing. We have offered ways of applying circular questions and have recommended using them especially when the instructor (1) feels s/he would like to learn something new or explore something deeper, (2) feels that s/he is taking sides, (3) feels that s/he is moving into an oppositional stance to the learners, (4) wants to highlight different points of view among team members, and (5) wants to explore circularity among team members. We have highlighted that requirements such as creating a respectful and engaging learning environment and adopting a systemic mindset are ideally in place before using circular questions.

Notably, we do not consider circular questions a replacement for other debriefing methods. Within the framework of blended debriefing approaches [5, 6, 25, 48], we recommend circular question as one further instrument of the instructor’s debriefing toolbox to be used in combination with other instruments and integrated into an overall debriefing model, ideally the *Debriefing with Good Judgment Approach* [9]. This embeddedness is essential to avoid disadvantages or pitfalls of circular questions such as appearing unfamiliar and strange, triggering surprising responses which catch the instructor off guard, or leaving the learner feeling interrogated. If circular questions are used excessively, the instructor becomes impalpable to the learners who might get frustrated from lack of direction and disengage from the debriefing [52]. Learners will not only need to perceive the instructor as someone trustworthy but also as someone who is willing to share his or her thinking, point of view, and expertise [2, 9, 50]. Instructors are advised to balance questions and advocacies and maybe even preview circular questions as such.

So far, empirical research on circular questions is sparse and almost non-existent in the context of healthcare simulation debriefings [41]. Research is needed to analyze debriefing interactions and their relation to debriefing outcomes and to investigate the effectiveness of circular questions. Also, more work is required to explore how circular questions can be integrated into simulation instructor faculty development programs as a method of debriefing team interactions by exploring the mutual dependency of team members’ behavior and recurrent behavior patterns, generating new information, and fostering perspective taking.

We hope this introduction of circular questions in simulation-based training will stimulate interesting debriefings, more research on debriefings, and help to walk the talk of teamwork during debriefings.
